# Time-Varying Transition Probability Matrix Estimation and Its Application to Brand Share Analysis

**DOI:** 10.1371/journal.pone.0169981

**Published:** 2017-01-11

**Authors:** Tomoaki Chiba, Hideitsu Hino, Shotaro Akaho, Noboru Murata

**Affiliations:** 1 Department of Electrical Engineering and Bioscience, Waseda University, Shinjuku, Tokyo, Japan; 2 Department of Computer Science, University of Tsukuba, Tsukuba, Ibaraki, Japan; 3 Mathematical Neuroinformatics Group, National Institute of Advanced Industrial Science and Technology, Tsukuba, Ibaraki, Japan; Nankai University, CHINA

## Abstract

In a product market or stock market, different products or stocks compete for the same consumers or purchasers. We propose a method to estimate the time-varying transition matrix of the product share using a multivariate time series of the product share. The method is based on the assumption that each of the observed time series of shares is a stationary distribution of the underlying Markov processes characterized by transition probability matrices. We estimate transition probability matrices for every observation under natural assumptions. We demonstrate, on a real-world dataset of the share of automobiles, that the proposed method can find intrinsic transition of shares. The resulting transition matrices reveal interesting phenomena, for example, the change in flows between TOYOTA group and GM group for the fiscal year where TOYOTA group’s sales beat GM’s sales, which is a reasonable scenario.

## Introduction

Multivariate time series recording of actual phenomenon may have dynamics based on an intrinsic variable structure. In particular, we consider the transition probabilities among products such as beer, automobiles, and newspapers. The transition matrix describes the probability of a change from one state to another state. In concrete terms, the transition matrix characterizes the shifts in consumers’ preferences towards different products in terms of probability. In other words, we assume that the (*i*, *j*)-element of the transition matrix is the probability that a consumer who previously bought the *i*-th product now purchases the *j*-th product instead.

Information on changes in consumers’ product purchases is necessary for understanding competition between products in the market. The intrinsic structure of transition matrices, however, cannot be directly observed because individuals’ purchasing data are typically difficult to obtain. Individuals’ data are also difficult to handle because of privacy issues. Owing to these issues, marketing data are often limited to product sales amounts such as point-of-sales data, aggregated by removing personally identifiable information. Therefore, a method to estimate and analyze the structure based on observations is indispensable for understanding hidden dynamics such as product share.

The method proposed in this paper estimates the transition matrices of customers switching between products by using only aggregated sales share data. Although it is impossible to uniquely determine the transition matrix from sales share data only, by making some natural assumptions on the transition matrix, we propose a method to estimate all of the transition matrices for every observation.

For the purposes of the mathematical formulation, we introduce the terminology of graph theory [1]. We identify products as nodes, and the relations between products are expressed by the edges between nodes. Consumers are supposed to move between nodes under the condition that the total number of consumers in the entire graph before and after the movement is constant. This consumer behavior is thus modeled as a finite state Markov chain with the constraint that the total number of consumers is fixed before and after the state transition [2]. The finite state Markov model is popular and widely used, for example, for modeling implied volatility in financial engineering [3], the distribution of individuals in community ecology [4], the distribution of the urban scale in demography [5], and the importance of websites, which is known as the PageRank model [6–8].

Suppose we observe non-negative multivariate time series such as the sales amount of different products. Let us normalize the observed non-negative multivariate time series data so that the sum of all variables is equal to one at each observation. Then, the normalized multivariate *π*_*t*_ at each observation time *t* is assumed to be the stationary distribution of the consumers on nodes. The stationary distribution $\pi_{t}\in R^{n}$ is a probability vector, which characterizes a transition matrix *G*_*t*_ at time *t* as $\pi_{t}^{⊤}G_{t}=\pi_{t}^{⊤}$. Then, we consider the problem of estimating the transition matrices corresponding to the observed stationary distributions.

In real problems, it is natural to assume that the intrinsic structure or consumers’ preferences vary over time. It is also naturally assumed that the transition probability varies at a slow pace. Additionally, we assume that an observation at time *t* is the stationary distribution of a transition probability matrix *G*_*t*_ at time *t*. Hence, we assume that the transition speed of the Markov chain induced by *G*_*t*_ is sufficiently fast.

The analysis of the relationship between variables in multivariate time series is of practical importance in many scientific fields and is also used in social and business data analysis [9–12]. Typically, to analyze the intrinsic structure between variables, it is useful to express the system as a graph composed of variables with nodes and edges representing their relationship. To estimate the intrinsic structure in the variables, causality analysis is a standard approach [13, 14]. This approach is actively studied particularly in econometrics [15]. These methods have been successfully applied to many problems, although they rely on statistical tests for every combination of variables and are computationally demanding. These methods also require a large number of observations to construct a statistical model and to perform a statistical test based on the model. With an increasing number of variables, many computationally efficient methods for estimating the covariance structure have been developed. One representative method is the graphical lasso [16], which is based on a sparse regularization and optimization algorithm. By using the graphical lasso and its variants, methods of analysing the time-varying graph structure are proposed and applied to change point detection [17–19], for example. These methods assume that the observed data are realizations of multivariate Gaussian distributions and suffer from low estimation accuracy for non-Gaussian behaviors in real-world problems. These methods are also unable to identify the asymmetric relationships between the variables. To overcome these problems, we develop a method of estimating the transition matrix without making an assumption about the distribution of the underlying covariates. The main contributions of this study can be summarized as follows:

- We propose a method for estimating consumer transitions between products at any moment by using sales share data only. By using our method, we can avoid the detailed recording of consumer transitions, which is high in cost or even impossible in reality.
- We apply our method to analyze consumer transitions for automobiles and provide a way in which to infer the change in consumers’ preferences towards different manufacturers. The result is reasonable and explains actual social/market events.

## Materials and Methods

### Material

Data to be analyzed are automobile sales data from the year 2007 to the year 2015 from various countries in quarterly units for each manufacturer. In the automotive industry, the positioning or branding of each manufacturer would gradually change, and the market share is assumed to be in a stationary state. Namely, we consider two different timescales. Within the quarterly unit, the transition of the consumers’ preferences are sufficiently rapid, that is, the transition of the consumers’ preferences is assumed to be in the stationary state. On the other hand, for a longer timescale, the change in the consumers’ preferences is assumed to be slow, and the underlying graph structures would gradually change.

**Fig 1:**
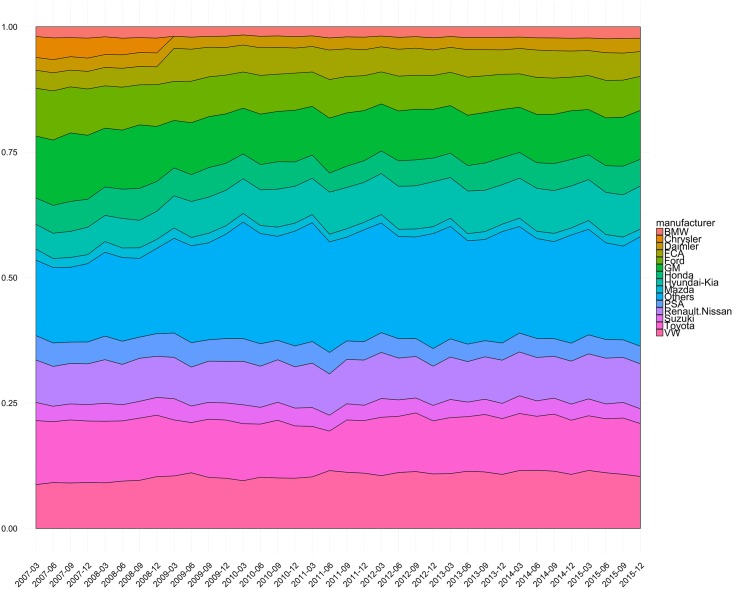
Quarterly sales share of manufacturers from the year 2007 to the year 2015.

For the sake of simplicity in analysis and visualization, among all manufacturers, the top 14 sellers (BMW Group, Chrysler Group, Daimler Group, FCA (Fiat Chrysler Automobiles), Ford Group, GM Group, PSA (Peugeot Société Anonyme), Renault-Nissan, VW Group, Suzuki, Toyota Group, Honda, Mazda, and Hyundai-Kia Group) are used singly, and other manufacturers are grouped and named “Others.” Then, the row sales data are transformed to the form of “sales share,” namely, the amount of sales is normalized to the ratio, which is regarded as a stationary distribution at a certain quarterly unit. The share data used in this paper are shown in Fig 1 as a stacked graph.

### Model Formulation

In this section, with some assumptions for changes in customers’ preferences, we model the relationship between the time series of the transition probability matrix and the share of products. Suppose there are *n* different products, and we observe a series of ratios or shares ${\left\{\pi_{t}\right\}}_{t=1}^{T}$, $\;\pi_{t}\in R^{n}$ of those products, namely, *π*_*t*_ represents the share of *n* products at time *t*. The ratio *π*_*t*_ satisfies conditions

$$\sum_{i=1}^{n}{\left(\pi_{t}\right)}_{i}=1,\;{\left(\pi_{t}\right)}_{i}\geq 0,\;\forall i\in \left\{1,\ldots ,n\right\}$$ (1)

where (⋅)_*i*_ is the *i*-th element of a vector. From the Perron-Frobenius theorem [20], *π*_*t*_ is considered an eigenvector of a stochastic matrix, namely, *π*_*t*_ is an eigenvector of a transition probability matrix. The transition probability matrix of consumers’ preferences on manufacturers at time *t* is denoted by $G_{t}\in R^{n\times n}$, where the (*i*, *j*) element of the matrix *G*_*t*_, which is denoted by (*G*_*t*_)_*ij*_, is the transition probability from the *i*-th product to the *j*-th product in a time interval (*t* − 1, *t*]. We assume that each element of the matrix *G*_*t*_ for all *t* ∈ {1, …, *T*} is strictly positive to account for the probability of random choice by consumers:

$$0<{\left(G_{t}\right)}_{ij}\leq 1,\;\forall i,j\in \left\{1,\ldots ,n\right\}.$$ (2)

Now our problem is estimating a set of transition matrices ${\left\{G_{t}\right\}}_{t=1}^{T}$ from observed ratios ${\left\{\pi_{t}\right\}}_{t=1}^{T}$, however, this estimation problem is typically indeterminate because the degree of freedom of ${\left\{G_{t}\right\}}_{t=1}^{T}$ is greater than that of observations ${\left\{\pi_{t}\right\}}_{t=1}^{T}$. Therefore, we need additional constraints on this problem. We impose the following two assumptions.

**Fig 2:**
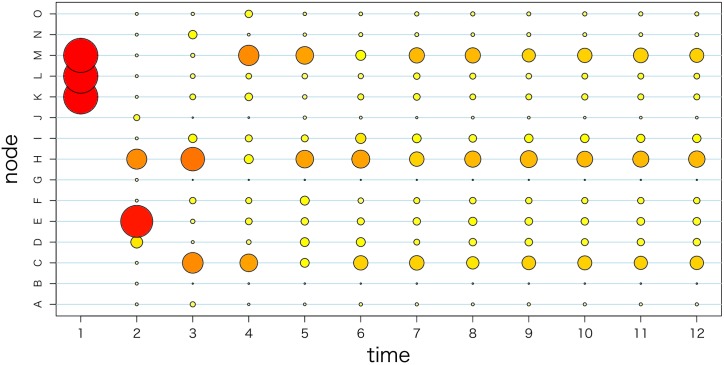
Example of the transition of distribution *π* through the actions of the transition matrix.

The first assumption is that the observed ratio *π*_*t*_ at time *t* is a realization of the stationary distribution of a Markov process represented by *G*_*t*_. Homogeneous Markov chain modeling based on the stationality at the observation interval has a long history in marketing research [21]. From research on PageRank, it is also acknowledged that the convergence of distribution *π*_*t*_, by the action of transition matrix *G*_*t*_, to the stationary distribution is very fast [22]. Fig 2 shows a simple example of the transition of distribution *π* by the consequent actions of transition matrix *G*. The number of nodes is set to 15 and we show the first 12 time steps of the sequence, namely {*π*, *Gπ*, *G*^2^*π*, …, *G*^11^*π*}. In this figure, the vertical axis shows the node number, which represents the elements of the vector $\pi \in R^{15}$. The sizes of the circles indicate the relative values of the elements. This figure shows almost no change in the distribution after time step *t* = 10. Because the Markov chain generated by transition matrix G is aperiodic, the stationary distribution is unique regardless of the initial value. In our model, we consider that the quarterly unit is sufficient for the observation to converge to the stationary distribution.

The second assumption is imposed on the time-varying property of transition matrix *G*_*t*_. Changes in consumers’ preferences towards each manufacturer are infrequent, and thus each element of *G*_*t*_ is similar to that of the previous transition matrix *G*_*t*−1_.

We summarize the assumptions on our model below. We treat a sequence of sales share, where each share is quarterly aggregated. We assume that transition matrix *G*_*t*_ in the *t*-th term is unchanged and that the observed sales share for the term well approximates the stationary distribution of the transition matrix. In other words, the timescale of the transition by the matrix is sufficiently small that the observed share at the end of the term have been converged to the stationary distribution. We note that the *transition* in the case of automobile share is not necessarily the actual purchase of cars by users because it is unusual to buy cars every three months. The transition here indicates the change in *preference* for brands by users, which affects users’ buying behavior.

It is also assumed that transition matrix *G*_*t*_ gradually changes and that the stationary distribution of the matrix also changes when observed at different terms. The difference between the consequent transition matrices is thus assumed to be small.

These assumptions about the model of the observed data and transition mechanism are mathematically embodied in the following section, and the problem of estimating the transition matrices is formulated as a simple linear programming.

### Optimization Problem for Estimating Transition Matrices

We derive a method for estimating a series of transition matrices ${\left\{G_{t}\right\}}_{t=1}^{T}$ corresponding to a series of observations ${\left\{\pi_{t}\right\}}_{t=1}^{T}$ of sales shares. We introduce the objective of optimization for estimating *G*_*t*_ and several constraints, which embody the assumptions stated in the previous section.

**Assumption 1 (Small and Sparse Change in the Transition Probabilities)** *It is natural to assume that changes in transition probability in consecutive observations are not so large. Specifically, we assume that the difference in each element of two consecutive transition matrices is small in terms of the* *ℓ*_1_-*norm and define the objective function to be minimized as follows:*

$$L\left({\left\{G_{t}\right\}}_{t=0}^{T}\right)=\sum_{t=1}^{T}{\left\|G_{t}-G_{t-1}\right\|}_{1},$$ (3)

*where* ‖*A*‖_1_, $A\in R^{n\times n}$ *is defined by* $\sum_{i,j=1}^{n}\left|{\left(A\right)}_{ij}\right|$.

It is worth noting that the *ℓ*_1_-norm minimization induces a sparse solution [23, 24], and the objective function (3) is called the *fused lasso* in the literature of sparse regularized regression [25]. We also note that we have to include $G_{0}\in R^{n\times n}$ for the target of estimation owing to this assumption. Since *G*_0_ does not have corresponding observation of share, we estimate *G*_0_ but do not give any interpretation for this extra transition matrix.

**Condition 1 (Stationary Distribution at Each Observed Time)** *The observed product share vector* *π*_*t*_ *is assumed to be the stationary distribution with respect to the transition matrix* *G*_*t*_, *which is formally expressed by the following condition:*

$$\pi_{t}^{⊤}G_{t}=\pi_{t}^{⊤},\;\forall t\in \left\{1,\ldots ,T\right\}.$$ (4)

**Condition 2 (Constraint to be a Transition Probability Matrix)** *By definition, the transition probability matrix is strictly positive. In addition*, *the following equations must be satisfied for* *G*_*t*_ *to be a transition matrix:*

$$\left\{\begin{array}{c} G_{t}e=e,\\ {\left(G_{t}\right)}_{ij}>0, \end{array}\right.\;\forall t\in \left\{0,1,\ldots ,T\right\}$$ (5)

*where* **e** *is an n dimensional vector with all ones*.

#### Mathematical Programming

Putting together the introduced objective function and constraints, we obtain the following optimization problem:

$$\underset{\left\{G_{t}\right\}}{\text{minimize}}\sum_{t=1}^{T}\left|\right|G_{t}-G_{t-1}{\left|\right|}_{1}\;\;s.t.\;\;\left\{\begin{array}{c} \pi_{t}^{⊤}G_{t}=\pi_{t}^{⊤},\;\forall t\in \left\{1,\ldots ,T\right\}\\ \begin{array}{c} G_{t}e=e,\\ {\left(G_{t}\right)}_{ij}>0. \end{array}\;\forall t\in \left\{0,1,\ldots ,T\right\} \end{array}\right.$$ (6)

#### Optimization

The optimization problem (6) is an instance of linear programming and is efficiently solved using the simplex method or the interior point method [26]. Namely, by introducing an auxiliary matrix $\Xi_{t}\in R^{n\times n}$, we can reformulate the problem (6) as linear programming:

$$\underset{\left\{G_{t}\right\}\left\{\Xi_{t}\right\}}{\text{minimize}}\sum_{t=1}^{T}\sum_{i,j=1}^{n}{\left(\Xi_{t}\right)}_{ij},$$ (7)

$$s.t.\left\{\begin{array}{cc} \begin{array}{c} \pi_{t}^{⊤}G_{t}=\pi_{t}^{⊤},\\ -{\left(\Xi_{t}\right)}_{ij}<{\left(G_{t}-G_{t-1}\right)}_{ij}<{\left(\Xi_{t}\right)}_{ij}, \end{array} & \;\forall t\in \{1,\ldots ,T\}\\ \begin{array}{c} G_{t}e=e,\\ {\left(G_{t}\right)}_{ij}>0. \end{array} & \;\forall t\in \{0,1,\ldots ,T\} \end{array}\right.$$ (8)

The algorithmic description of the proposed method is shown in Algorithm 1.

**Algorithm 1** Algorithm for estimating the transition matrices

 **Input**: Non-negative multivariate time series.

 **Initialization**: Normalized the observed time series to a sequence of stationary distributions ${\left\{\pi_{t}\right\}}_{t=1}^{T}$.

 **Estimation**: Solve the linear programming Eq (7).

 **Output**: Estimated sequence of transition matrices ${\left\{G_{t}\right\}}_{t=1}^{T}$.

## Results and Discussion

**Fig 3:**
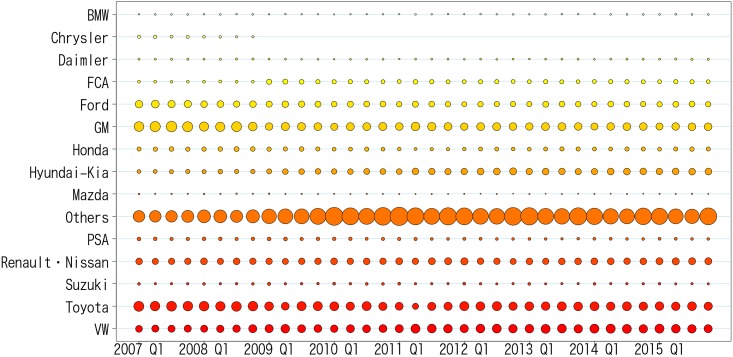
Observed sales share data of manufacturers from the year 2007 to the year 2015.

**Fig 4:**
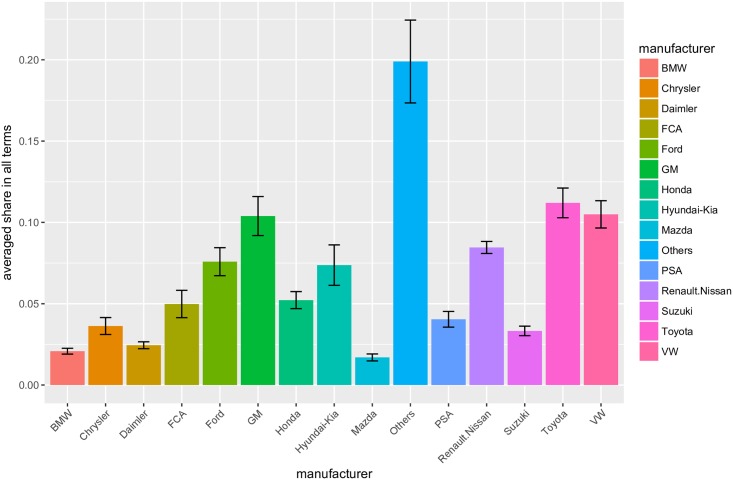
Averages and standard deviations of the sales shares.

For the quarterly unit automobile sales data of manufacturers from 2007-1Q to 2015-4Q, we performed the prepossessing explained in the Materials and Methods. We note that 1Q, 2Q, 3Q, and 4Q denote the first, second, third, and fourth quarter in a fiscal year. Then, we applied the proposed method for a series of observed share data ${\left\{\pi_{t}\right\}}_{t=1}^{T}$, where *t* = 1 corresponds to “2007-1Q,” and *t* = *T* corresponds to “2015-4Q,” to estimate a series of transition probability matrices. The optimization problem (7) is solved by the simplex method using the solver for linear programming GLPK [27]. The observed data is shown in Fig 3, which expresses the same information shown in Fig 1. Fig 1 is popular for showing market information, while Fig 3 is more intuitive in terms of market share transition for a certain time unit. The size and color of circles indicate the proportion of automobile sales for different manufactures. Fig 4 shows the averages and standard deviations of the sales shares in all terms. This figure shows that the standard deviations of shares tend to be large for manufacturers with a large market share. Further, the Renault-Nissan group has a relatively high average and small standard deviation, which indicates that this manufacturer maintains a certain market share stably. On the contrary, the Hyundai-Kia group has a relatively low average but a large standard deviation. This fact suggests that this manufacturer is growing rapidly (see also Fig 3). While we can infer the above-mentioned facts and tendencies from the market share data, it is impossible to identify how the consumer transitions from one manufacturer to another with sales share data alone. In the following subsections, we show the graphs representing the estimated transition paths with discussion on and consideration for social events that may explain the estimated results.

### Estimated Transition Matrices and Corresponding Market Structure

**Fig 5:**
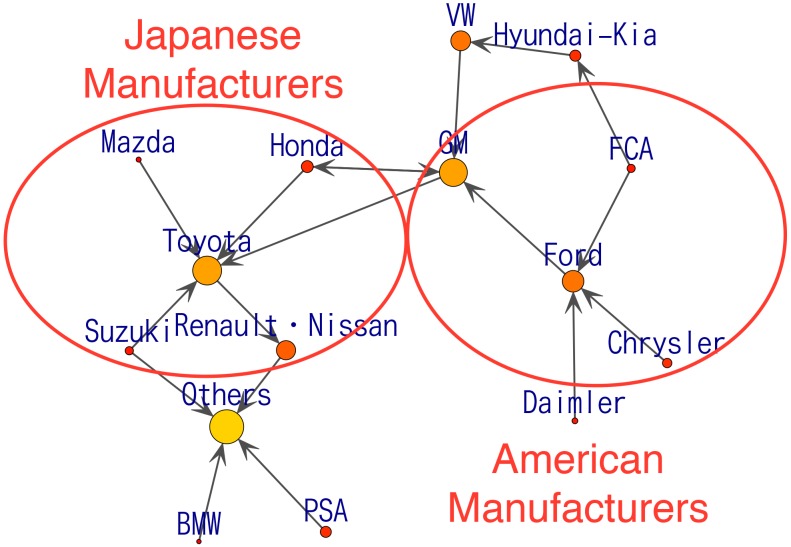
The estimated directed graph for “2007-1Q”.

Transition matrices are constrained to be positive and there are flows of customers from any manufacturer to any other manufacturer. We hereafter set elements of the estimated transition matrices below certain threshold to zero to remove minor edges for visualization purpose. The threshold used to visualize the results in this paper is 0.24, which offers legible results. Fig 5 shows the estimated transition matrix *G*_1_ obtained by solving the linear programming Eq (7), and the corresponding directed graph of automobile manufacturers’ sales shares for the first term (“2007-1Q”) of all records. The size of the circle at each node represents the market share of the corresponding manufacturer. The transition matrix is asymmetric. The (*i*, *j*) element of matrix *G*_*t*_ denoted by (*G*_*t*_)_*ij*_ is the transition probability from the *i*-th product to the *j*-th product in the time interval (*t* − 1, *t*]. We draw an arrow from the *i*-th node to the *j*-th node with a width proportional to the magnitude of (*G*_*t*_)_*ij*_ in the graph, representing the transition matrix at time *t*. In the directed graph, arrows connecting the nodes indicate that there are flows of sales share or flows of customers in the direction indicated by the arrows. Bi-directed arrows indicate that both connected nodes have in/out flows. The results show two distinct groups. One group includes American manufacturers such as Chrysler Group, Ford Group, GM Group, and the other group includes Japanese manufacturers such as Suzuki, Honda, TOYOTA Group, Mazda, and Renault-Nissan. Interestingly, there is a bi-directed arrow between GM and Honda, which are alliance companies. This phenomenon is presumably because the car dealer of each manufacturer recommends the cars of the alliance partner to customers. It is also possible that the same car dealer sells both GM and Honda cars.

### Explanation of Social Events Inferred from the Transition Matrix

**Fig 6:**
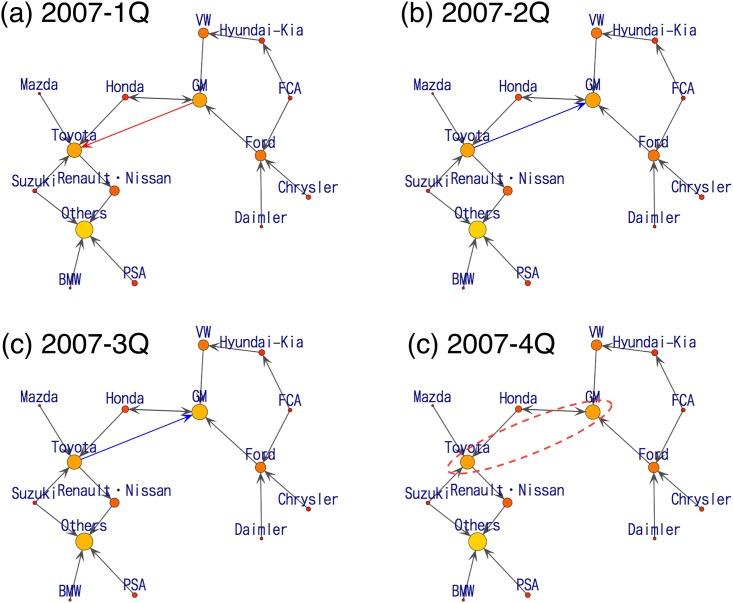
Visualization of consumers’ flow estimated from a series of data from year 2007. There is a remarkable change in the direction and presence of the arrow between TOYOTA Group and GM Group.

We present a more detailed analysis. First, within the fiscal year ending March 2008, TOYOTA Group has become the world’s top seller by beating GM Group in unit sales. We show the estimation results from “2007-1Q” to “2007-4Q” in Fig 6. From Fig 6(a), a direct flow from GM Group to TOYOTA Group is observed at 2007-1Q (red arrow). Then, for the next two quarterly periods shown in Fig 6(b) and 6(c), we infer that TOYOTA Group’s activity caused a reaction, and the graph shows an arrow from TOYOTA Group to GM Group (blue arrow). After this fluctuating behavior, at the fiscal year end in December shown in Fig 6(d), we do not see salient movement in consumers between those two manufacturers.

**Fig 7:**
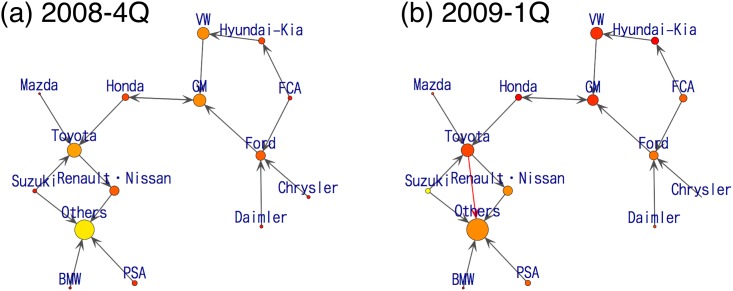
Visualization of estimated consumers’ flow from a series of data in 2008-4Q and 2009-1Q.

We next focus on 2009-1Q in Fig 7. In 2009, TOYOTA Group launched a massive recall and decreased its share to a large extent. This can be seen by comparing the size of the circles for TOYOTA Group in 2008-4Q shown in Fig 7(a) and in 2009-1Q shown in Fig 7(b). Comparing the graphs in 2008-4Q shown in Fig 7(a) and 2009-1Q shown in Fig 7(b), we see that from 2009, there is an arrow from TOYOTA Group to Others (red arrow), which indicates the defection of customers.

**Fig 8:**
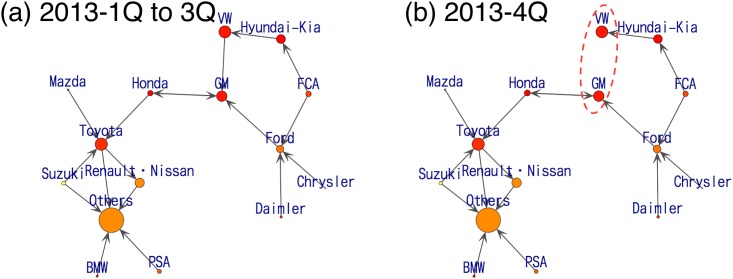
Visualization of estimated consumers’ flow from a series of data in year 2013.

Finally, we focus on the year 2013 (Fig 8). In the second half of this year, VW Group beats GM Group in total sales amount to claim second position in the automobile industry. From Fig 8(a) and 8(b), the consumers’ flow from VW Group to GM Group disappears in the 4th quarter in 2013, which indicates improvements in the brand image of VW compared to GM.

## Conclusion

### Summary of Contribution

In this paper, we considered the situation that manufacturers compete for limited consumers. By modeling the transition of consumers between different manufacturers using a Markov chain, we proposed a method to infer a sequence of transition matrices of consumers to different manufacturers using a sequence of sales share data only. In the proposed model, the observed sales share data are identified with stationary probabilities of underlying Markov processes characterized by transition matrices. Assuming that the change in the structure, namely, a change in the transition matrices for consequent time is minor, we formulated the estimation problem of transition matrices as simple linear programming. The proposed method is applied to sales data for automobiles, and we obtained reasonable and socially explainable results. We believe that the results are significant in the sense that we can infer the flow of consumers only from sales share data. The results can be utilized for a market analysis or to develop a brand strategy with limited observations. For illustrative purposes, we considered the transition of consumers among manufacturers. However, the proposed method is applicable to more general situations with a fixed amount of *resources*, which is an abstraction of consumers, and nodes compete for finite resources through the edges.

### Future Work

To estimate the transition matrices, we imposed necessary constraints so that the estimates should be transition matrices. Under these constraints, we minimized absolute difference between consequent matrices. There are other possibilities for optimizing objectives and constraints to improve the accuracy of estimation and interpretability. It would also be interesting to directly model the change in transition matrices with appropriate probability models.

## Supporting Information

S1 Code Program code and dataset to reproduce the results. Python code for the proposed method, and original dataset are available as a supporting information file.
(ZIP)
